# Supplementary material for: Antizyme Inhibitor 2-Deficient Mice Exhibit Altered Brain Polyamine Levels and Reduced Locomotor Activity
Source: Biomolecules. 2022 Dec 21;13(1):14. doi: 10.3390/biom13010014 (PMC9855896; doi:10.3390/biom13010014)
Supplement: Supplementary file 1 [file biomolecules-13-00014-s001.zip › biomolecules-2088770-supplementary figures.pdf]

Supplementary material

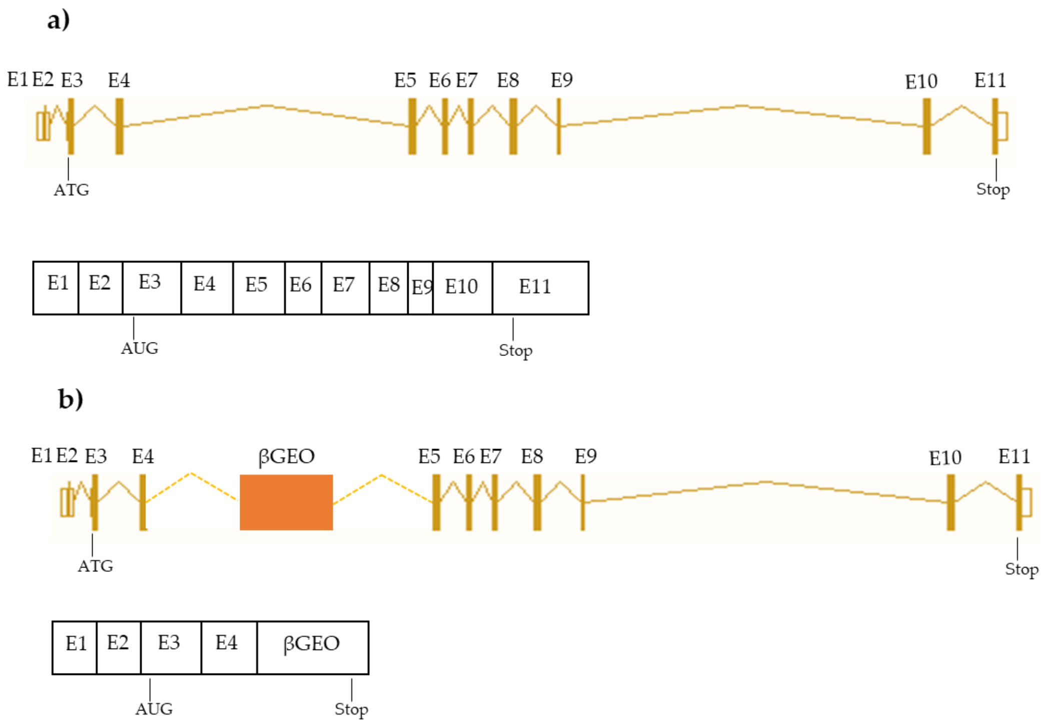

Figure S1. (**a**) Genomic structure of *mAzin2* gene (WT). (**b**) Genomic structure of *mAzin2*$^{\beta Geo}$.

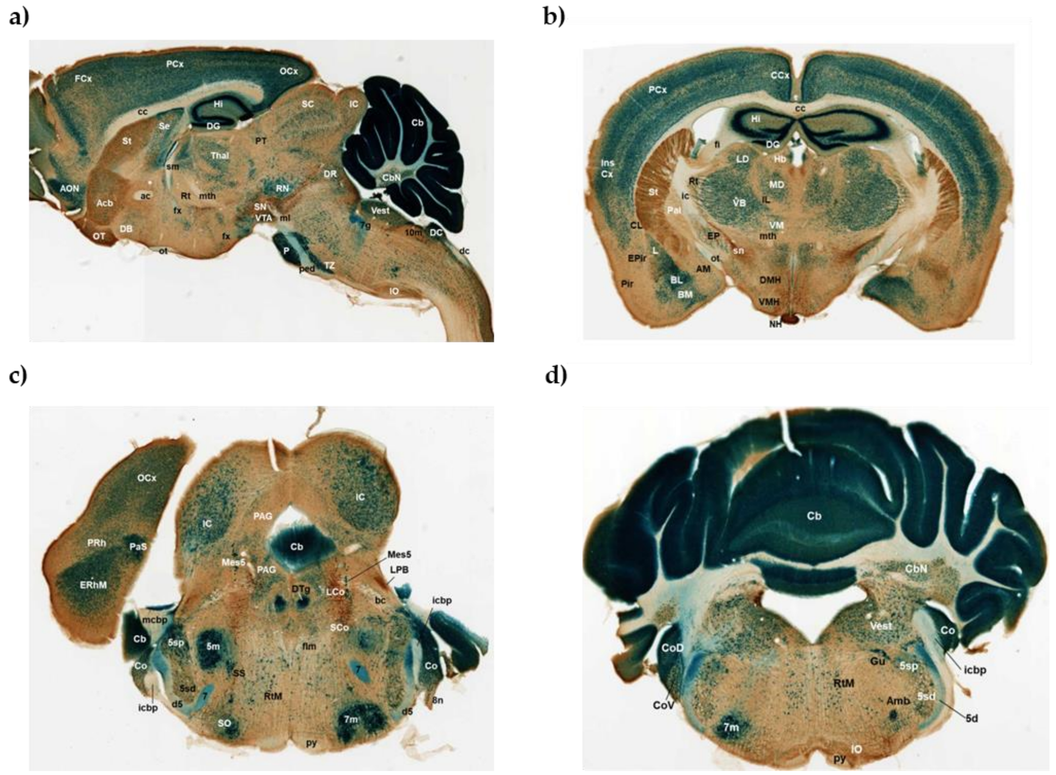

Figure S2. Histological sections of KO brains stained with X-gal (blue) and counter-stained with tyrosine hydroxylase (brown) in (**a**) Sagittal, and (**b**–**d**) coronal sections of KO brains. 10 m, vagus nerve; 5 m, motor trigeminal nerve (r5); 7g, genu of the facial nerve; 7 m, facial motor nucleus; ac, anterior column; Acb, Accumbens; AM, amygdala; AON, anterior olfactory nucleus (retrobulbar region); BL, basolateral nucleus; BM, basomedial amygdaloid nucleus; Cb,

cerebellum; CbM, medial cerebellar nucleus; cc, corpus callosum; CCx, cerebral cortex; CL, claustrum; Co, commissural nucleus; CoD, dorsal cochlear nucleus; CoV, ventral cochlear nucleus; Cx, cortex; DB diagonal band; DC, dorsal claustrum; dc, dorsal column; DG, dentate gyrus; DMH, dorsomedial hypothalamic nucleus; DR, dorsal raphe nucleus; DTg, dorsal tegmental 2nucleus; EP, entopeduncular nucleus; FCx, frontal cortex; fl, frontal lobe; flm, medial longitudinal tract; fx, fornix; Gu, gustatory nucleus; Hb, habenula; Hi, hippocampus; hy, hippothalamus; IC, internal capsule; icbp, inferior cerebellar peduncle; IL/L, intermediolateral/lateral solitary subnucleus; Ins, insula; IO, inferior olive; Lco, locus coereleus; LD, laterodorsal thalamic nucleus; mcbp, medial cerebellar peduncule; MD, medial dorsal thalamic nucleus; Mes5, trigeminal mesencephalic nucleus; ml, medial lemniscus; mth, mammillo-thalamic tract; P, basilar pontine nuclei; PAG, periaqueductal gray; Pal, paralaminar nucleus; PaS, parasubiculum; ped, cerebral peduncle; Pir, parainsular region; PRh; PT, paratenial thalamic nucleus; py, pyramidal tract; RN, red nucleus; Rt, reticular thalamic nucleus,; RtM, medial (basal) reticular formation; SC, superior colliculus; sm, stria medullaris of thalamus; SN, substantia nigra; SO, superior olivary formation; SS, straight sinus; St, stria terminalis; Thal, thalamus; TZ, trapezoid body decussation; Vest, vestibular nucleus; VM, ventromedial thalamic nucleus; VMH, ventromedial hypothalamic nucleus; VTA, ventral tegmental area.

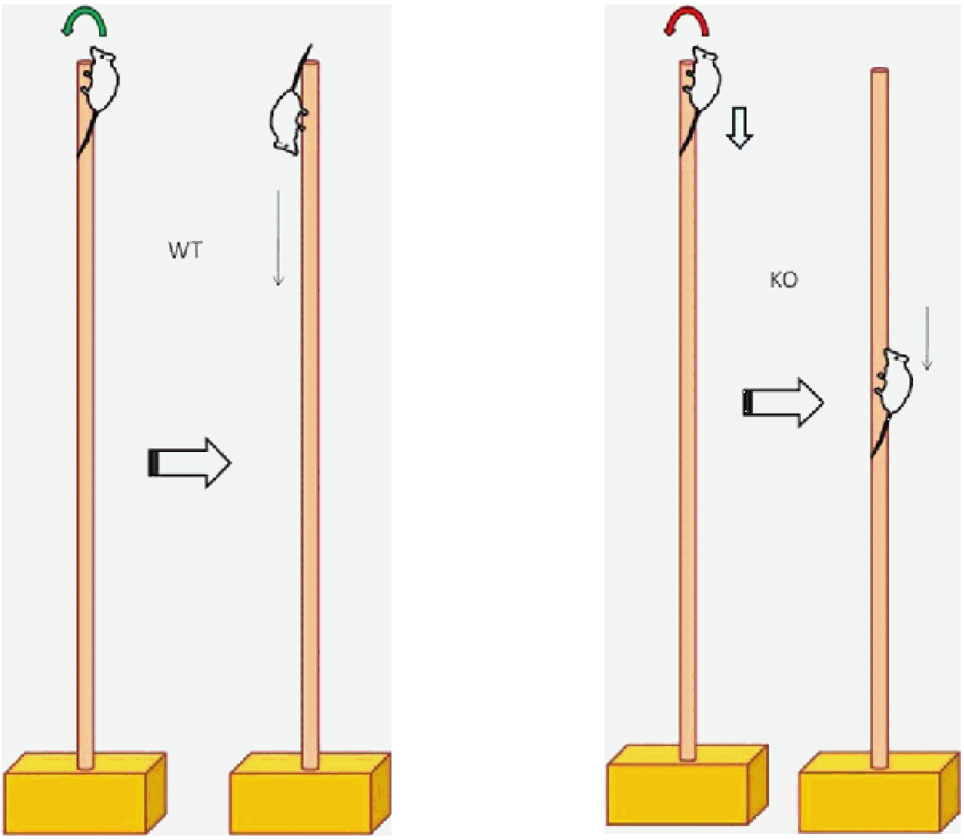

Figure S3. Pole test scheme and representation of the results obtained in the WT and KO groups.
