# Supplementary material for: Antizyme Inhibitor 2-Deficient Mice Exhibit Altered Brain Polyamine Levels and Reduced Locomotor Activity
Source: Biomolecules. 2022 Dec 21;13(1):14. doi: 10.3390/biom13010014 (PMC9855896; doi:10.3390/biom13010014)
Supplement: Supplementary file 1 [file biomolecules-13-00014-s001.zip › biomolecules-2088770-supplementary tables.pdf]

Supplementary Table S1. List of primers.

Gene Name	Official Gene Symbol[a]	Forward (5′-3′)	Reverse (5′-3′)
Ornithine decarboxylase, structural 1	*Odc1*	ATGGGTTCCAGAGGCCAAA	CTGCTTCATGAGTTGCCACATT
Ornithine decarboxylase antizyme 1	*Oaz1*	GAGTTCGCAGAGGAGCAACT	CCAAGAAAGCTGAAGGTTCG
Ornithine decarboxylase antizyme 2	*Oaz2*	AGTAAGTGTCCCCAGCTCCA	ATCTTCGACAGTGGGTGAGG
Antizyme inhibitor 1	*Azin1*	CTTTCCACGAACCATCTGCT	TTCCAGCATCTTGCATCTCA
Antizyme inhibitor 2	*Azin2*	GCTTAGAGGGAGCCAAAGTG	CTCAGCAAGGATGTCCACAC
β-actin	*Actb*	GATTACTGCTCTGGCTCCTAGCA	GCTCAGGAGGAGCAATGATCTT
β-galactosidase	*LacZ*	TTATCGATGAGCGTGGTGGTTATGC	GCACGATAGAGATTCGGGATTT

[a] Mouse Genome Informatics Database.

Supplementary Table S2. Ornithine carboxylase activity during the postnatal period.

Time (days)	mmol $^{14}CO_2$ h^{-1} g^{-1} (mean ± SE)
**1**	5.864 ± 0.635
**2**	4.583 ± 0.278
**5**	4.117 ± 0.316
**7**	2.235 ± 0.452
**10**	0.905 ± 0.123
**20**	0.070 ± 0.009
**60**	0.078 ± 0.012
